# Stomal Hyperflow and Intestinal Failure: A Case Report on the Use of a Chyme Reinstillation Device

**DOI:** 10.7759/cureus.91493

**Published:** 2025-09-02

**Authors:** Florian Poncelet, Thierry Van Der Linden, Sabrina Dardenne, Edouard-Florent Pourbaix, Emmanuel Floret

**Affiliations:** 1 Pharmacy, Hôpital Saint Philibert Lomme, Lomme, FRA; 2 Intensive Care Medicine, Groupement des Hôpitaux de l'Institut Catholique de Lille, Lille, FRA; 3 Digestive Surgery, Hôpital Saint Vincent de Paul, Lille, FRA

**Keywords:** chyme reinstillation, intestinal failure, medical devices, nutritional disorders, stomal hyperflow

## Abstract

Stomal hyperflow, a frequent complication of ileostomy, is associated with high morbidity and mortality, particularly when linked to intestinal failure. This situation, especially when conventional treatments fail, often results in a poor prognosis for the patient.

In this case report, we present the history of a patient suffering from stomal hyperflow with intestinal failure, in the context of short small bowel syndrome following multifocal venous mesenteric ischaemia. In a critical condition at the time of admission to our hospital, he was able to benefit from The Insides® System, a chyme reinstillation device, with the aim of improving his general condition and ultimately enabling surgical restoration of digestive continuity.

This system led to clinical and biological improvement, allowing surgery to restore continuity. However, certain difficulties related to the use of the device were also encountered during management.

## Introduction

In France, it is estimated that around 100,000 patients live with an ostomy. There are three main types: urological stomas, digestive stomas for nutritional purposes, and digestive stomas for faecal diversion. There are two main types of stomas: colostomies, where the colon is surgically connected to the abdominal wall, and small intestine stomas, which involve connecting parts of the small intestine to the abdomen, such as jejunostomies, where the jejunum is connected to the abdominal wall, and ileostomies, where the ileum is connected to the abdomen [1].

Although few precise data are available on the exact number of patients with ileostomies in France, a study carried out in the United Kingdom estimates that 64,000 people have them, representing around 36% of the country’s ostomy population [2].

One of the most common complications of ileostomy is stomal hyperflow, defined as a volume of more than 1.5 L/d for two consecutive days [3], compared with a normal flow rate of 600 mL to 1.2 L/d [1]. Occurring in over 30% of cases, it is associated with significant morbidity [4]. The causes are varied: infections, drugs, or mechanical causes such as short small bowel syndrome [1]. This condition can lead to a longer hospital stay, with rehospitalisation in 17% of cases, as well as nutritional complications [1]. The most common clinical consequences include dehydration, acute renal failure, undernutrition, and fluid and electrolyte disturbances [5].

Treatment of stomal hyperflow is based on three principles: restriction of hypotonic fluids to avoid worsening the flow, and use of anti-motility and antisecretory drugs to limit digestive secretions [6].

In some cases, this hyperflow may be accompanied by intestinal failure, particularly after extensive resection of the small intestine. This corresponds to an inability of the intestine to absorb nutrients, water, and electrolytes sufficiently, necessitating prolonged parenteral nutrition until surgical resumption of digestive continuity [7]. It requires complex multidisciplinary management and intravenous supplementation for periods of up to several months or years [8]. Associated mortality varies between 6% and over 50%, depending on the study and the presence or absence of complications [9-11].

If conventional treatments fail, solutions such as surgical restoration or chyme reinstillation may be considered [1]. Reinstillation involves restoring functional continuity between the two intestinal segments. Recommended by the European Society of Clinical Nutrition and Metabolism, this technique is particularly indicated in cases of double enterostomies or high-flow fistulas. It makes it possible to re-establish enteral nutrition and gradually wean the patient off parenteral nutrition [8]. Traditionally performed with continuous pumps (now obsolete), it can now be carried out with new devices, such as The Insides® System (The Insides® Company, New Zealand), which operate intermittently by bolus [12]. However, these devices are still new and not widely used. The main reasons for this are the barriers to using these devices, both in terms of regulations and clinician training. These obstacles can limit access to this technology and, consequently, its use. For its integration into clinical practice to become a reality, it will therefore be necessary to anticipate the difficulties associated with its implementation.

The aim of this case report is to present the use of The Insides® System in a situation encountered in our hospital. We will detail its implementation, management procedures, and the clinical benefits observed in the patient.

Written informed consent was obtained from the patient for publication of this case report and any accompanying images. Permission was also obtained from The Insides® Company to use the device name, brand name, and images of the device.

This case was presented in abstract form and as part of an oral presentation at the 2024 International Conference on Clinical Case Reports on 10 October 2024.

## Case presentation

A man aged between 75 and 80, who had undergone a double bowel resection with two double-barrel stomas (jejunal and ileal), performed following multifocal venous mesenteric ischaemia, was admitted to intensive care for stomal hyperflow. On admission, he was found to have severe protein-energy malnutrition, loss of muscle mass and strength, dehydration, and metabolic acidosis.

**Figure 1:**
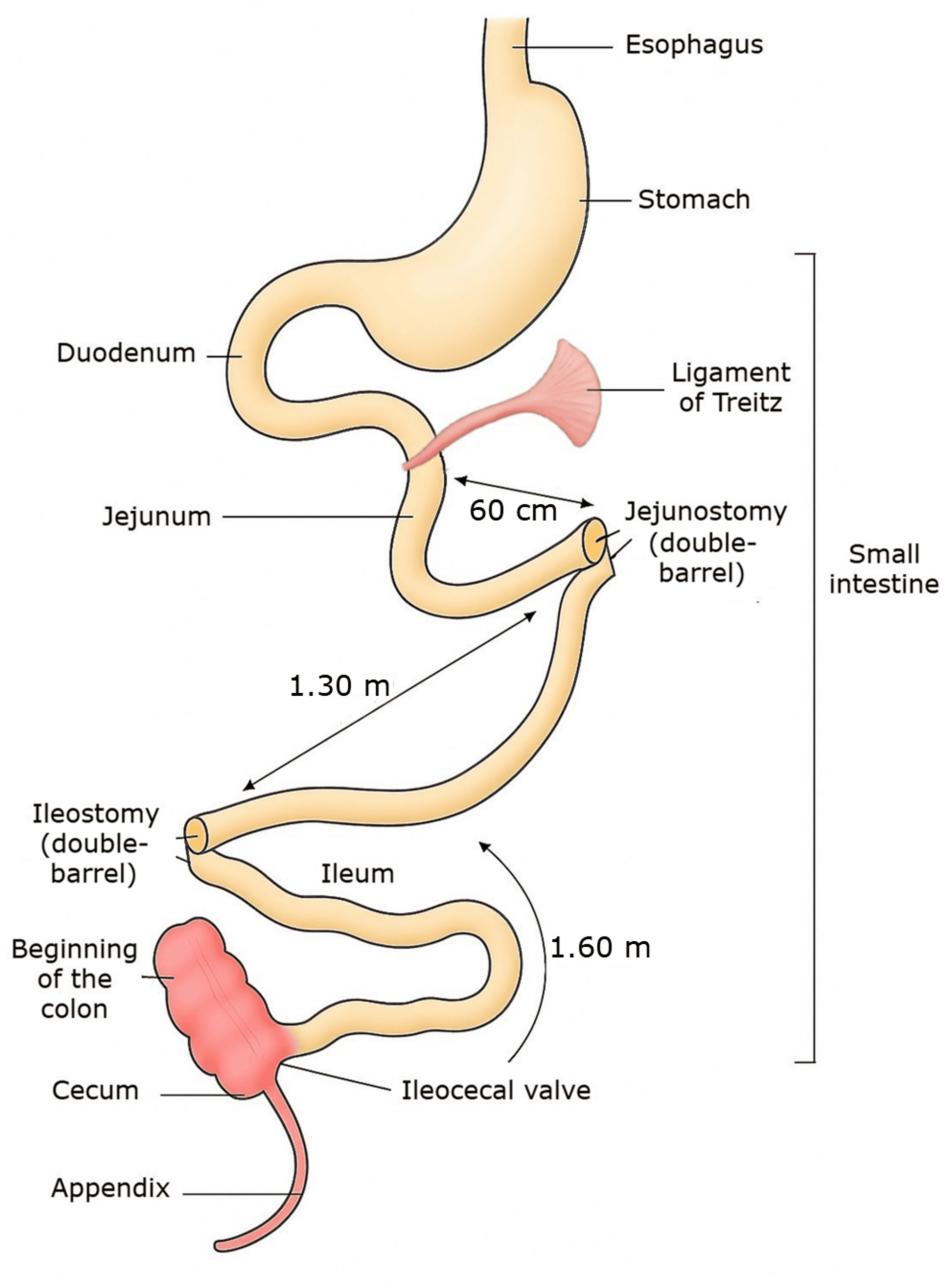
Diagram showing the anatomical position of the two stomas in the patient’s small intestine. Figure generated using artificial intelligence (ChatGPT/OpenAI) and refined with Pixlr software to optimise scientific presentation.

Prior to this hospitalisation, surgical follow-up of the mesenteric ischaemia had led to attempts to restore continuity of the two stomas, which were successful for the jejunal stoma but failed for the ileal stoma. More specifically, regarding the locations of these stomas: at 60 cm from Treitz's angle (the duodenojejunal junction suspended and held in place by Treitz's ligament), a 10 cm area of jejunal necrosis was resected and a stoma was created, while 1.30 m downstream, extensive ileal necrosis required the resection of 1.50 m and the creation of a second stoma. The remainder of the distal small intestine remained healthy, i.e. 1.60 m up to the ileocecal valve (Figure 1).

**Figure 2:**
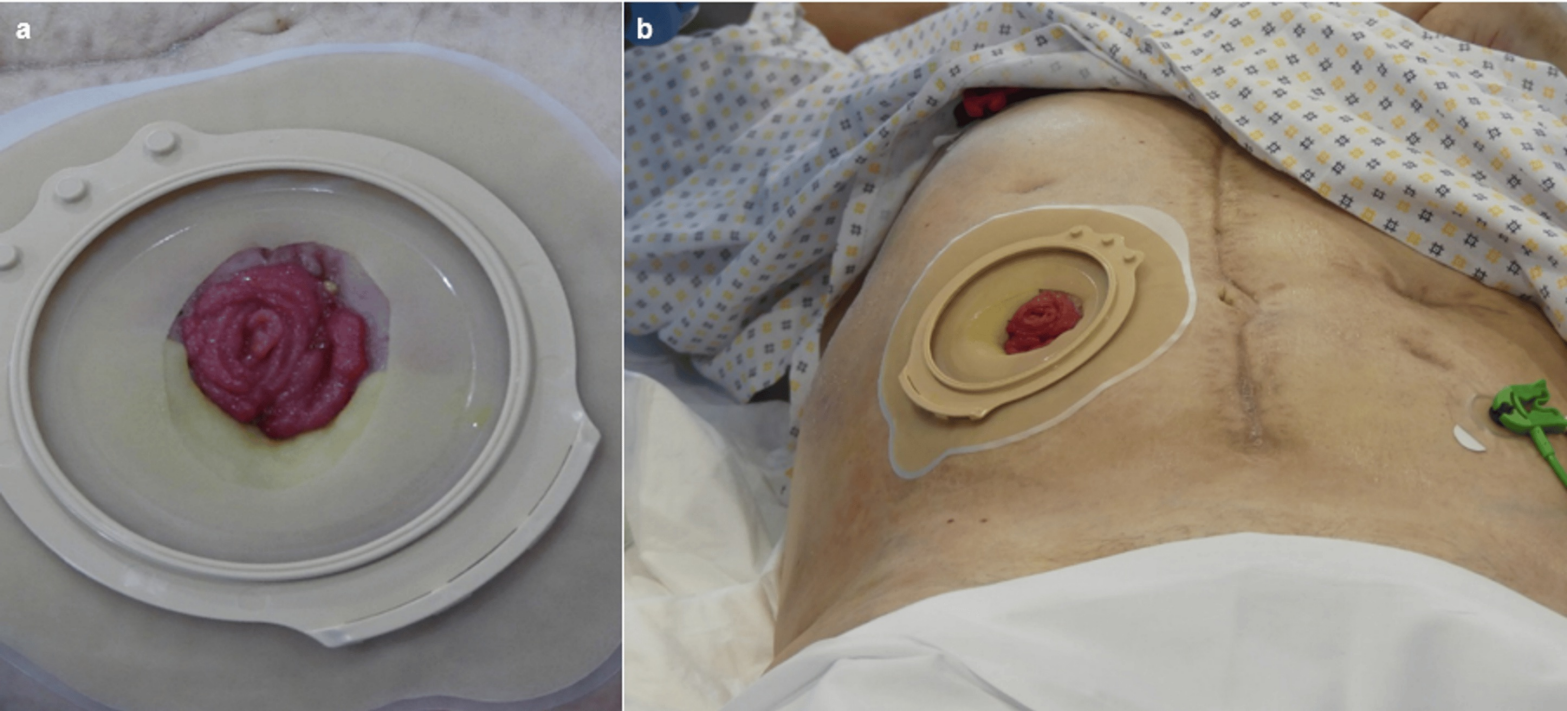
Photographs showing the ileal stoma (a) and its anatomical location (b). Written informed consent was obtained from the patient.

The first attempt to re-establish digestive continuity in the ileal stoma, where stomal hyperflow was already present, was unsuccessful, as a loose suture led to an anastomotic fistula and generalised peritonitis (Figure 2).

In addition to the patient’s chronic and anti-infectious treatments, he was also placed on enteral and parenteral nutrition at the start of his hospitalisation, due to his critical clinical condition. Conventional treatments for stomal hyperflow were attempted, but without success. Furthermore, the stomal hyperflow produced a large and sudden output, making stoma fitting particularly difficult, with frequent detachment of the pouches.

**Figure 3:**
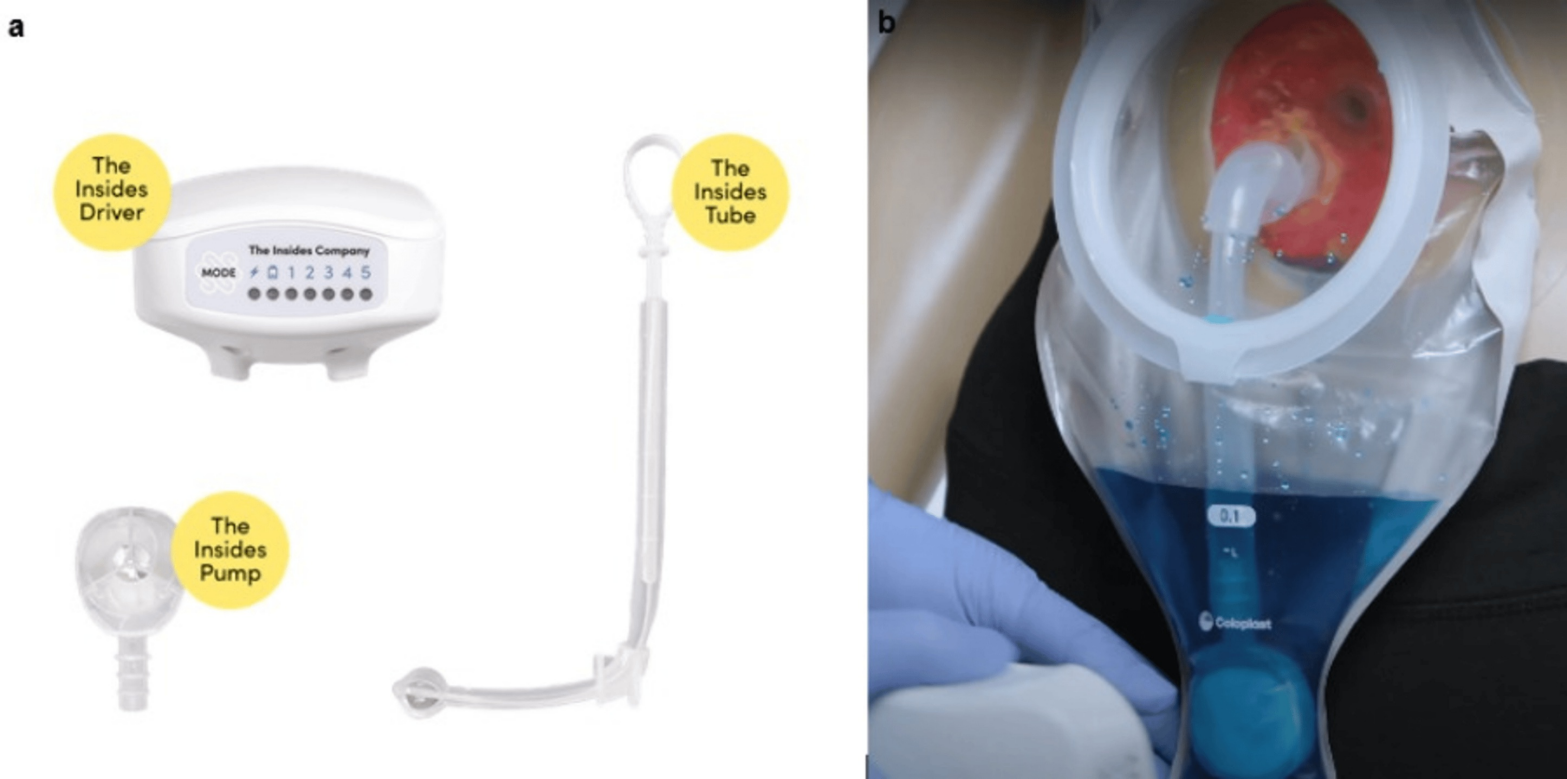
Description of the components of The Insides® System (a) and photograph showing insertion of the device with an ostomy pouch (b). Permission to use these images was obtained from the manufacturer (The Insides® Company).

Faced with these therapeutic failures, the patient was implanted with a chyme reinstillation system in the stoma (The Insides® System, The Insides® Company, New Zealand). This device consists of a feeding tube inserted into the intestine, a pump connected to this tube, and an electromagnetic controller. Chyme is collected directly from the ostomy pouch and then reinjected intermittently by bolus into the downstream intestinal segment (Figure 3).

The aim of this device was to stabilise the patient’s nutritional status by maintaining a degree of functional continuity of transit, in order to prepare him for the surgical restoration of digestive continuity, while limiting the risk of refistulisation associated with severe malnutrition.

Before installing the device, a number of checks had to be carried out to confirm its feasibility for this patient. As the system consisted of consumables that had to be changed frequently (every month for the catheter and every two or three days for the pump and the ostomy pouch), the supply chain was verified. The average time between ordering and receiving consumables was around 10 days.

Patient eligibility was also assessed, based on inclusion and exclusion criteria, particularly the absence of infectious signs and severe psychiatric disorders, as well as radiological verification of the absence of stenosis in the downstream digestive segment. Once these elements had been validated, the device could be fitted.

**Figure 4:**
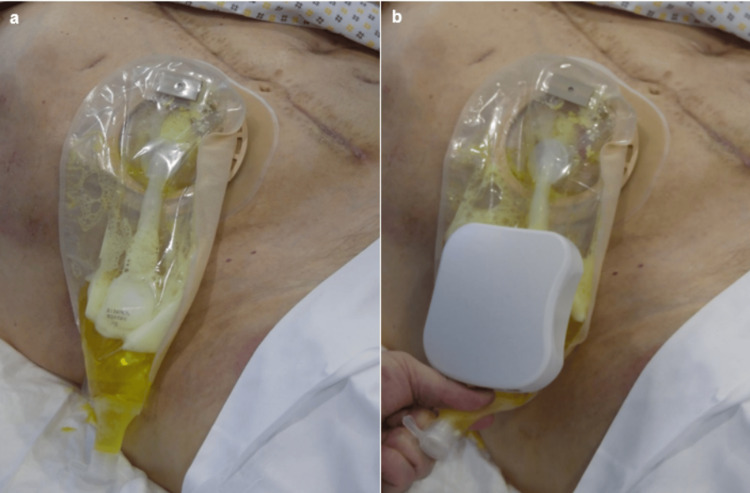
Photographs showing installation of the device (a) and the motor test to verify correct operation (b). Written informed consent was obtained from the patient.

The procedure was carried out without difficulty or pain. Once the device was in place, a motor test was performed to ensure that the system was functioning properly, with conclusive results (Figure 4).

Following insertion of the device, the patient benefited from post-operative monitoring and discontinuation of transit-slowing drugs to avoid chyme becoming too thick to be reinstilled. No changes were made to the dosage of his chronic medications. Reinstillation was carried out every 2 to 3 hours, depending on the volume and viscosity of the chyme, for optimum efficacy.

A significant improvement was observed within the first few days. Stomal losses, initially estimated at between 1.8 and 2 L/d, fell to less than 500 mL/d. This evolution was reflected in a marked improvement in clinical condition, enabling a rapid reduction in parenteral nutrition requirements (from 1.5 L/d to 1 L/d), while maintaining oral nutritional supplements. The favourable evolution of the patient’s general condition enabled his transfer from the ICU to the digestive surgery unit, and then discharge to hospital at home.

In total, the patient benefited from this device for 44 days prior to the restoration of small intestinal continuity. We noted an increase in weight of 3 kg in 4 weeks (BMI from 23.1 to 24.1 kg/m²), muscle strength (grip test from 18 kg to 21.6 (right) and <10 kg to 18.1 (left)), muscle mass (muscle mass index from 7.22 to 8.53 kg/m²: an improvement of 18%), and a correction of hydration and acid-base balance.

Biological changes during use of the device showed, in particular, an improvement in albumin (31%) and pre-albumin (84%). There was also a significant reduction in proteinuria (30%) and C-reactive protein (50%) (Table 1).

**Table 1:** Changes in the main biological parameters before device installation and at the time of device removal. (↑: increase; ↓: decrease).

Biological parameters studied	Usual values of the establishment	Time of installation of the device	Time of withdrawal	Evolution
Albumin	35-50 g/L	19.9 g/L	26 g/L	↑ 31%
Prealbumin (Transthyretin)	200-400 mg/L	107 mg/L	197 mg/L	↑ 84%
Potassium	3.3-4.8 mmol/L	3 mmol/L	3.4 mmol/L	↑ 13%
Sodium	136-145 mmol/L	134 mmol/L	136 mmol/L	↑ 1%
Creatine phosphokinase	39-308 U/L	26 U/L	78 U/L	↑ 200%
Creatininemia	6.5-11.7 mg/L	11.1 mg/L	11.9 mg/L	↑ 7%
Magnesium	16-26 mg/L	15.8 mg/L	21.2 mg/L	↑ 34%
Phosphorus	25-49 mg/L	34.4 mg/L	36.6 mg/L	↑ 6%
Chloride	98-107 mmol/L	104 mmol/L	104 mmol/L	0%
Proteins (urinary)	< 0.10 g/L	0.83 g/L	0.58 g/L	↓ 30%
C-reactive protein	< 8 mg/L	32 mg/L	16 mg/L	↓ 50%

Tolerance was good. The main problem was persistent leakage of ileal fluid, causing skin burns, related to the anatomical position of the stoma (close to the iliac crest), the patient’s morphology (significant peristomal segmental adipose-muscular wasting causing an anatomical hollow), and poor patient compliance with stoma care. Other obstacles, such as the need for training and user protocols, frequent pump changes, and management constraints, were also observed [12].

## Discussion

Managing stomal hyperflow refractory to conventional treatments is a significant clinical challenge, often associated with rapid deterioration in patient condition, especially when accompanied by intestinal failure. In this case report, the use of a chyme reinstillation system demonstrated substantial clinical and biological improvements, particularly in nutritional and hydration status, enabling the patient to undergo surgical restoration of digestive continuity under optimal conditions. No technical malfunctions of the device were reported, and complications encountered, such as stoma leaks, were unrelated to the system itself.

The principle of chyme reinstillation involves restoring intestinal continuity by redirecting proximal stoma effluent into the distal bowel. This process has been shown to significantly improve fluid and nutrient absorption and reduce dependence on parenteral nutrition. These benefits are well documented in the literature. In particular, the 15-year prospective cohort study by Picot D et al., which at the time used a device operating with continuous pumps, reported a significant reduction in stoma output, improved fat absorption, and increases in weight, BMI, and albumin, enabling successful weaning from parenteral nutrition [13].

In a communication presented at the 2022 Francophone Nutrition Days, The Insides® System demonstrated superior nutritional intake, better tolerance, and improved quality of life compared to the continuous pump system, although it required frequent boluses [14]. However, its use entails several prerequisites: patient and caregiver education, clear protocols for use and maintenance, thorough staff training, and rigorous logistical management of consumables, which must be replaced frequently and may pose a practical constraint [12].

One of the main strengths of our report is the comprehensive longitudinal follow-up, from device insertion to removal, which allows for a detailed assessment of clinical benefits and operational challenges. In addition, the multidisciplinary management of the case, involving pharmacists, intensive care physicians, digestive surgeons, and other professionals, enabled a detailed and comprehensive analysis.

Although data remain limited due to the recent launch of the device, initial studies are encouraging [14-16]. Compared with these studies, our report adds value by incorporating more detailed clinical parameters, such as muscle mass and strength, rather than focusing solely on weight gain, and by openly discussing practical limitations and drawbacks, which are often underestimated. Furthermore, while Farrer K et al. reported that more than a third of patients did not tolerate the reinstillation device, our patient tolerated the system without any problems, highlighting the variability of patient responses and justifying further research [16].

The encouraging outcomes observed have contributed to the establishment of a reimbursement system for this device in France, which should facilitate wider clinical adoption and support the collection of additional real-world data [12].

Future prospective studies involving larger cohorts are needed to confirm these benefits, optimise protocols for use, and assess patients’ long-term tolerance and quality of life. In addition, continuous improvements to the device, particularly aimed at reducing logistical constraints, will be essential for its sustainable integration into the management of this type of patient.

## Conclusions

This case demonstrates that The Insides® System can effectively treat refractory stomal hyperflow associated with intestinal failure, leading to significant improvements in nutritional and hydration status, and enabling rapid surgical restoration of digestive continuity. Contrary to previous reports of frequent intolerance, our patient tolerated the device well, highlighting its potential for individualised clinical application. Although operational constraints, such as patient education and consumables management, remain challenges, the demonstrated efficacy and safety of the device justify its consideration as a valuable transitional therapy. The recent establishment of reimbursement frameworks further supports its integration into clinical practice. Larger-scale studies with this device will be necessary to validate these results, optimise protocols, and evaluate long-term outcomes. Nevertheless, this case illustrates the substantial clinical benefit that this chyme reinstillation device can offer in the complex management of such patients.
